# Tactile display of softness on fingertip

**DOI:** 10.1038/s41598-020-77591-0

**Published:** 2020-11-24

**Authors:** Gabriele Frediani, Federico Carpi

**Affiliations:** Department of Industrial Engineering, University of Florence, Via di S. Marta, 3, 50139 Florence, Italy

**Keywords:** Engineering, Materials science

## Abstract

Multi-sensory human–machine interfaces are currently challenged by the lack of effective, comfortable and affordable actuation technologies for wearable tactile displays of softness in virtual- or augmented-reality environments. They should provide fingertips with tactile feedback mimicking the tactual feeling perceived while touching soft objects, for applications like virtual reality-based training, tele-rehabilitation, tele-manipulation, tele-presence, etc. Displaying a virtual softness on a fingertip requires the application of quasi-static (non-vibratory) forces via a deformable surface, to control both the contact area and the indentation depth of the skin. The state of the art does not offer wearable devices that can combine simple structure, low weight, low size and electrically safe operation. As a result, wearable softness displays are still missing for real-life uses. Here, we present a technology based on fingertip-mounted small deformable chambers, which weight about 3 g and are pneumatically driven by a compact and cost-effective unit. Weighting less than 400 g, the driving unit is easily portable and can be digitally controlled to stimulate up to three fingertips independently. Psychophysical tests proved ability to generate useful perceptions, with a Just Noticeable Difference characterised by a Weber constant of 0.15. The system was made of off-the-shelf materials and components, without any special manufacturing process, and is fully disclosed, providing schematics and lists of components. This was aimed at making it easily and freely usable, so as to turn tactile displays of softness on fingertips into a technology ‘at fingertips’.

## Introduction

A major goal for the next generation of multi-sensory human–machine interfaces is the mimicry of the sensation of touching virtual objects that are soft. Physically displaying the softness of a computer-generated structure is essential to enable a diversity of virtual- or augmented-reality systems for various possible uses. Examples include simulators for training of medical professionals in the palpation of soft tissues^1^, tele-operation systems^2^, computer-aided design^3^, 3D model exploration^4^, as well as tele-presence systems for social interactions augmented by the sense of touch^5^.

Mimicking with high accuracy and realism the tactual feeling produced by the indentation of a soft object with a fingertip requires haptic displays. They are interfaces that should provide users with ideally both kinds of information that our brain integrates to perceive softness: tactile feedback and kinaesthetic feedback. The former is related to temporal and spatial variations of the contact pressure and contact area between the fingertip and object, as well as displacements of their surfaces^6–8^. The latter engages the position and velocity of the joints of the arm and forces of its muscles^6–8^.

Nevertheless, Srinivasan and LaMotte have demonstrated that, for the perception of the softness of objects having a deformable surface (and not just being compliant with a rigid surface), tactile feedback alone is sufficient, whilst kinaesthetic feedback alone is insufficient^7^. This has been interpreted as due to the fact that, for any applied force, the object’s compliance determines the deformation of the fingertip’s skin, and therefore it can adequately be encoded by cutaneous mechanoreceptors^7^. This is consistent with later evidences on the importance of the variation of the contact area between fingertip and object^9–11^, such that the change in contact area has been proposed as a new proprioceptive cue^12^.

Therefore, the softness of virtual objects cannot be rendered using the various existing haptic displays that provide purely kinaesthetic feedback (e.g. see those reviewed in^13^). The simplest effective approach is to rely on purely tactile feedback, using so-called tactile displays.

Moreover, in order to allow users to freely move their hands while performing a virtual- or augmented-reality task, the tactile displays should be wearable, i.e. sufficiently small and light to be arranged on fingertips^13^. Therefore, the tactile perception mode should be of the kind usually referred to as ‘passive’, where the fingertip does not move with respect to the whole device and is deformed by the actuation of an interface.

Developing such wearable devices able to create realistic tactile feedback requires, however, a clarification on the role of different tactile cues that interplay in the perception of softness. Although this is still a matter of discussion today^14^, Dhong et al. have recently shown that, in addition to the contact area, also the indentation depth is an essential tactile stimulus and they independently concur to the perception of softness^15^. So, tactile displays that control only the contact area or the indentation depth are expected to be less effective than devices that could control both of them^15^.

As a consequence, softness rendering cannot effectively be achieved with the variety of wearable tactile displays that apply forces to fingertips via stiff surfaces, which are however very performing in generating force feedback, especially for shape rendering^16–21^. Indeed, such displays just control indentations of the skin and, as discussed by Srinivasan and LaMotte, for an interface that generates a force via a rigid surface, the pressure distribution over the fingertip and the associated deformation of the skin (and so also the contact area) are independent of the interface’s compliance; therefore, the tactile stimuli gathered by the mechanoreceptors in this situation are unable to adequately encode information on compliance^7^.

According to these evidences, the most effective strategy to physically render the softness of a virtual object is to use tactile displays able to deliver quasi-static (non-vibratory) forces, via a soft interface (deformable surface), so as to control both the contact area and the indentation depth.

In order to obtain such devices, an explored strategy is to use rotary motors that drive flexible/stretchable structures (e.g. polymer membranes or fabrics), which however typically lead to complex, bulky and heavy mechanisms^22,23^. To overcome those drawbacks, an alternative approach is to use soft materials (elastomers) in a way that allows them to be deformed without complex mechanical transmissions from an actuation source. Three technologies under investigation in that direction are dielectric elastomer actuators (DEAs), electrostatic actuators and pneumatic actuators.

DEAs, which consist of electrically deformable elastomeric membranes^24^, have been used for non-vibratory fingertip displays suitable for softness rendering, adopting buckling^25,26^, cone^27^ and hydrostatically coupled^28,29^ configurations. The main limitation of using DEAs for tactile displays is the current need for high driving voltages, which requires challenging developments^30^. Similarly, high driving voltages are a drawback for purely electrostatic actuators^31^.

In contrast, electrically safe, compact and lightweight interfaces on fingertips can be obtained with pneumatic actuation, as the driving source can be displaced remotely. So far, it has been used for wearable devices in three ways: the first one employs air jets via an array of nozzles, which however limit the realism of the experience, due to the lack of a soft interface^32,33^; the second one is based on pneumatically driven rigid pins, which however serve, as discussed above, for shape rather than softness rendering^34^; the third strategy uses inflatable chambers, which appear as a simple and effective approach^35–37^.

Recently, Sonar et al. have demonstrated a small fingertip-mounted chamber, which was also able of self-sensing, proposed for closed loop control^38^. However, due to the entirely soft structure of the chamber, pressurisation causes outwards bulging, reducing the effect that actuation has on the finger pulp. So, it was not designed to preferentially control the indentation depth and contact area, and indeed it was particularly performing in transmitting dynamic forces, for vibratory feedback^38^.

Furthermore, this and all previous works on pneumatic tactile displays have not addressed the need for compact (truly portable) and low-cost pneumatic driving units, as well as the possible use of off-the-shelf materials and simple manufacturing processes for the whole system, so as to ease fabrication and lower costs. Those unmet needs have practically prevented so far a wide spread of pneumatic driving for tactile displays, despite its potential^39^.

Due to such a lack of simple and affordable enabling technologies, applications of softness displays to real-life systems have yet to come.

Here, we describe pneumatically-driven fingertip displays of softness, which combine effective functionality with a simple structure, low weight and low size, not only of the wearable interface but also of its driving unit. In order to make it easily and freely usable, the whole system was intentionally developed using only off-the-shelf materials and components, without any special manufacturing process, and it is here fully disclosed with schematics and lists of components.

## Results and discussion

### Structure and principle of operation of the softness display

**Figure 1:**
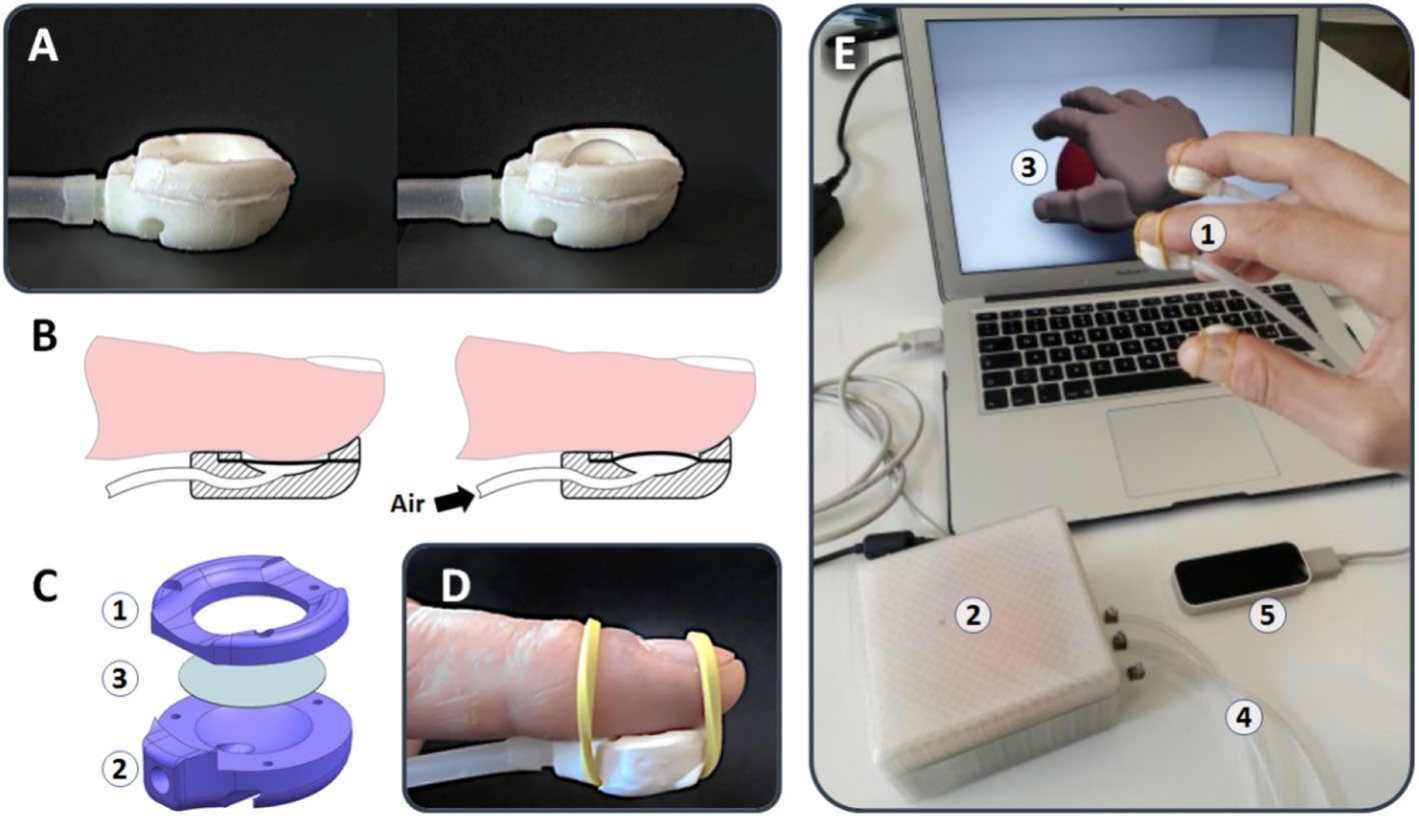
Illustrations of the softness display and example of a possible use. (**A**) Optical images of a prototype display, showing a deformation of the membrane upon pressurisation. (**B**) Sectional schematic drawings showing the principle of operation of the device as a display of softness: pressurisation deforms the membrane, so as to cause an indentation of the fingertip’s skin and an increase of the contact area, which are two essential tactile stimuli concurring to the perception of softness. (**C**) Exploded drawing of the device, showing that it consists of two plastic parts (1)–(2), which sandwich and laterally constrain an elastomeric membrane (3). (**D**) Optical image of the prototype display secured to a fingertip by means of elastic bands; the weight of the display was 3 g. (**E**) Example of use of the device: three fingertip-mounted displays (1) are simultaneously and independently controlled by the electro-pneumatic unit (2), to render the softness of a computer-generated deformable ball (3); connections to the unit are via thin elastomeric tubing (4), stretchable and not just flexible, so as to minimise impact on finger movements; the latter are monitored in real time by an optical sensor (LEAP Motion, USA) arranged below the hand (5); information captured by the sensor is used to continuously detect the intended motor action (extent of ball squeezing) and return both a visual feedback on the screen and a related tactile feedback on the fingertips.

The device is shown in Fig. 1. It consists of a small plastic chamber, which is closed by a thin elastomeric membrane and can be pressurised with air, in order to deform the membrane.

At rest, the membrane is flat and closes a rounded cavity, which hosts an opening through which air can flow in and out, for pressurisation and depressurisation (Fig. 1). The plastic chamber has an ergonomic shape, in order to comfortably conform to adult finger pulps. By securing the device to a fingertip (e.g. by means of elastic bands, as in Fig. 1), the actuation of the membrane can be used to indent the finger pulp and increase the contact area (as well as the transmitted total force). As recalled in the Introduction, these features make the display particularly suited to mimic contact with soft bodies.

An applied pressure *p* causes in the elastomeric membrane a stress *σ*, which drives its actuation. As the membrane behaves as a thin-walled spherical pressure vessel, the stress can be estimated as follows^40^:

1 $$\sigma =\frac{p R}{2d}$$

where *R* is the radius of curvature of the pressurised membrane and *d* is its thickness. The occurring deformation dynamically depends on the visco-hyper-elastic properties of both the membrane and the finger pulp, as well as the geometry of the latter, as commented later on in the text.

The prototype sample shown in Fig. 1 had a maximum length and thickness of 22.5 and 8 mm, respectively. The internal chamber had a diameter of 12 mm, which was chosen as a (non-optimised) trade-off between the needs for maximising the contact area with the fingertip (so as to maximise the perceivable force) and minimising the device encumbrance. That size was consistent with the average width of thumb and index finger pulps among participants to a psychophysical test described in the following. The display was reported as comfortable by all the subjects, in consideration of not only its shape but also its weight, which was just 3 g.

In order to ensure repeatability and simplify a possible adoption of this technology from others, the display was manufactured using a commercial PDMS membrane (see “Methods”), instead of preparing any custom sample from raw materials.

As the major drawbacks of pneumatic actuation systems are, in general, the size, weight and cost of the driving equipment, in this work we developed an electro-pneumatic control unit with a compact design and a low-cost architecture. The unit was based on a small pump, six valves and pressure sensors, as well as a microcontroller. It was USB connected to a personal computer, so as to be driven by any external software via serial communication. A custom script for the microcontroller was developed to control the pressure values of up to three fingertip displays independently.

The total weight of the control unit was 380 g, which made the technology easily portable, as shown in Fig. 1E. That figure presents a prototype implementation of the whole system, as well as an example of a possible use to physically mimic the softness of a virtual object, like a deformable ball visualised on a computer screen. This scenario is just mentioned here as an example of a low-cost virtual environment, whose realism can be enhanced by the proposed tactile technology in a simple and affordable manner. The tactile displays allow for rendering the tactual perception of squeezing the ball, while the screen returns a contextual visual representation of the action via an avatar hand interacting with the ball. To that end, the tactile display system is used in combination with a motion tracking sensor (commercially available desktop device), to detect the positions and movements of the fingers, i.e. the intended motor task on the ball. The sensor output is used to move the avatar hand and, at the same time, close the loop on the real hand, providing a related tactile feedback.

In order to make this technology freely and easily usable, all the information necessary to build the system is provided as “Supplementary Information”. This includes the files of the CAD drawings of the display structure, the electrical and mechanical schematics of the electro-pneumatic unit, as well as the list of the required electrical and mechanical components.

The following sections present physical and psychophysical characterisations of the system.

### Static and dynamic free stroke performance

**Figure 2:**
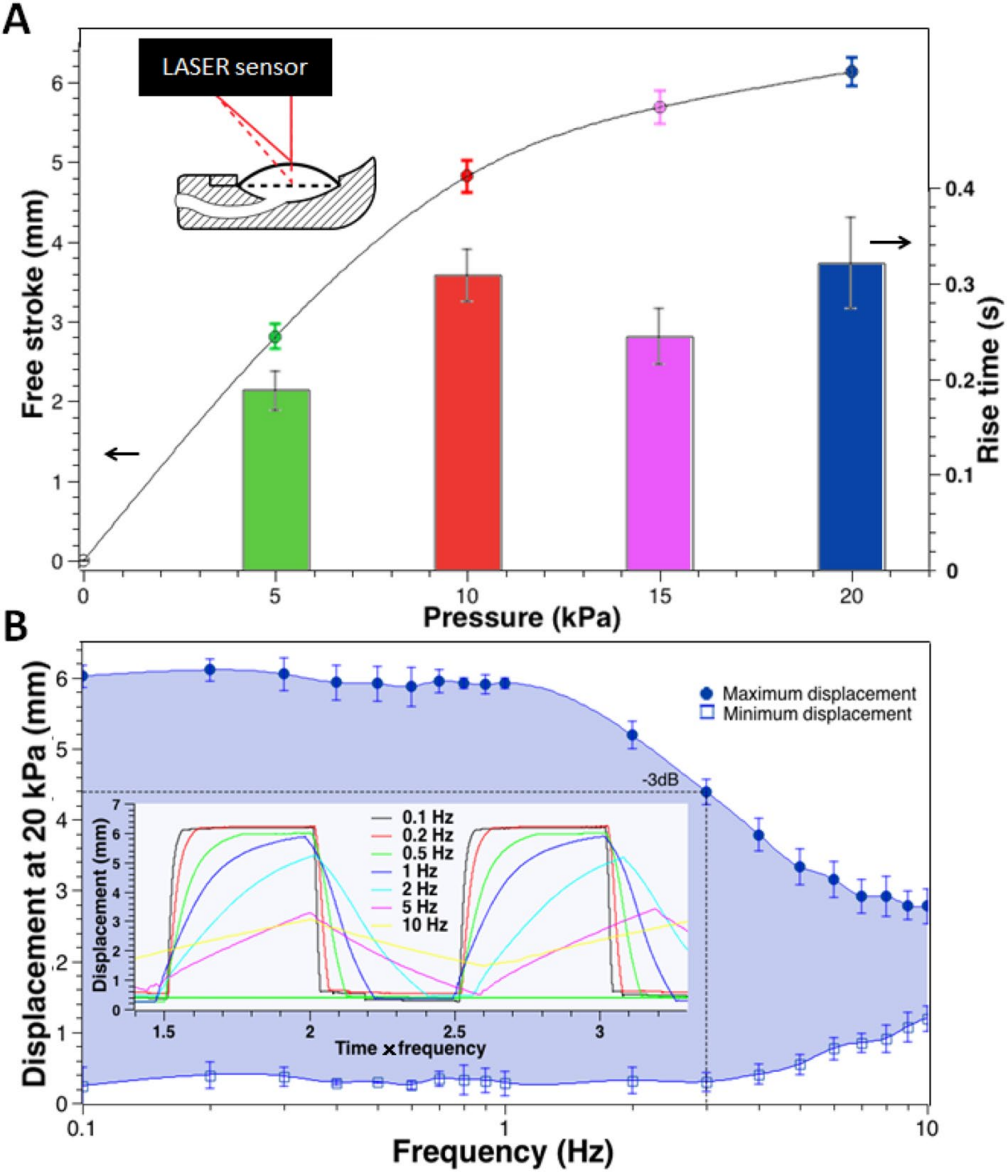
Static and dynamic free stroke performance of the softness display. (**A**) Static free stroke and rise time (free stroke response time) as functions of the driving pressure. (**B**) Dynamic free stroke as a function of the driving frequency: the frequency response for square pressure waves at 20 kPa is plotted in terms of maximum and minimum apical displacements; the inset graph shows examples of dynamic free stroke signals. The error bars represent the standard deviation among three different samples in (**A**) and (**B**), and also three different measurements for each sample in (**A**).

The tactile displays’ free stroke, defined as the apical (central point) displacement of the membrane without any applied load, was assessed in response to increasing pressures, up to 20 kPa, both statically and dynamically. The tests were performed with a LASER transducer, as detailed in “Methods”. The static free stroke performance is presented in Fig. 2A.

The maximum displacement at 20 kPa was about 6 mm (Fig. 2A). This value might be modified by changing the membrane’s compliance, according to its hyperelasticity and thickness. As expected, the free stroke was not linear with the pressure, due to the non-linearity introduced by both the hyperelastic behaviour of the membrane and its actuation mode.

In order to assess the reaction speed of the whole system (tactile display and driving unit) upon a step-wise pressure command, the membrane’s rise time, i.e. the free stroke response time, was calculated as the time required to displace the membrane’s apex from 10 to 90% of the steady-state free stroke (see Supplementary Fig. S1). Figure 2A reports the rise time for different pressures, showing that it varied between about 200 and 300 ms.

As an observation, it is worth stressing that the non-monotonic increase of the rise time with the pressure (see the drop at 15 kPa in Fig. 2A) was likely caused by the electromechanical behaviour of the valves. Indeed, whilst they were controlled with a proportional signal (see “Supplementary Information 1”), a non-linearity of their mechanical response altered the air flow, and so also the membrane’s rise time. Such behaviour could be fixed in the future with higher quality valves or improved strategies to drive them.

A thorough characterisation of the dynamic performance of the system was achieved by determining its frequency response with square pressure waves at 20 kPa, as detailed in “Methods”. The results are presented in Fig. 2B, which plots both the maximum and minimum apical displacements, as functions of the driving frequency. For the maximum displacement plot, the cut-off frequency, corresponding to a − 3 dB drop of the response from its low-frequency value, was about 3 Hz (Fig. 2B).

This bandwidth was determined by various factors, including, as a major contribution, the limited speed of the electro-pneumatic valves, as well as the inertia of the amount of air to be displaced, some losses due to friction, and the mass and visco-hyper-elastic properties of the membrane. Anyhow, a bandwidth of 3 Hz is considered sufficient for rendering a virtual softness. Indeed, the natural probing of an object’s compliance typically involves quasi-static motor tasks, where the motion dynamics usually do not exceed frequencies of the order of 1 Hz.

Furthermore, it is worth noting that the measurement of the bandwidth from free stroke actuation allowed for conservatively assessing the worst case scenario of use. Indeed, during the actual contact with a fingertip, the display never operates in free stroke mode. Similarly, it also never operates in blocking-force mode (characterised in the next section). This is due to the fact that, for any given driving pressure, the shape and deformability of a fingertip have on the display a certain loading effect (which varies among different individuals). Therefore, the display operates in variable intermediate conditions, between the free stroke and blocking force modes. In particular, with respect to a free stroke operation, the actual dynamic response is less affected (to an extent that depends on the contact area) by the mechanical limiting factors listed above. As a result, the response time measured from the free stroke tests over-estimates the actual delay in operative conditions.

### Indentation force and blocking force performance

The complementary information of the free stroke performance required to fully characterise the system is represented by its ability to generate forces that can be perceived by users. To gather that information, the force of the display was characterised with both objective tests (presented below) and subjective tests (presented in the next section).

**Figure 3:**
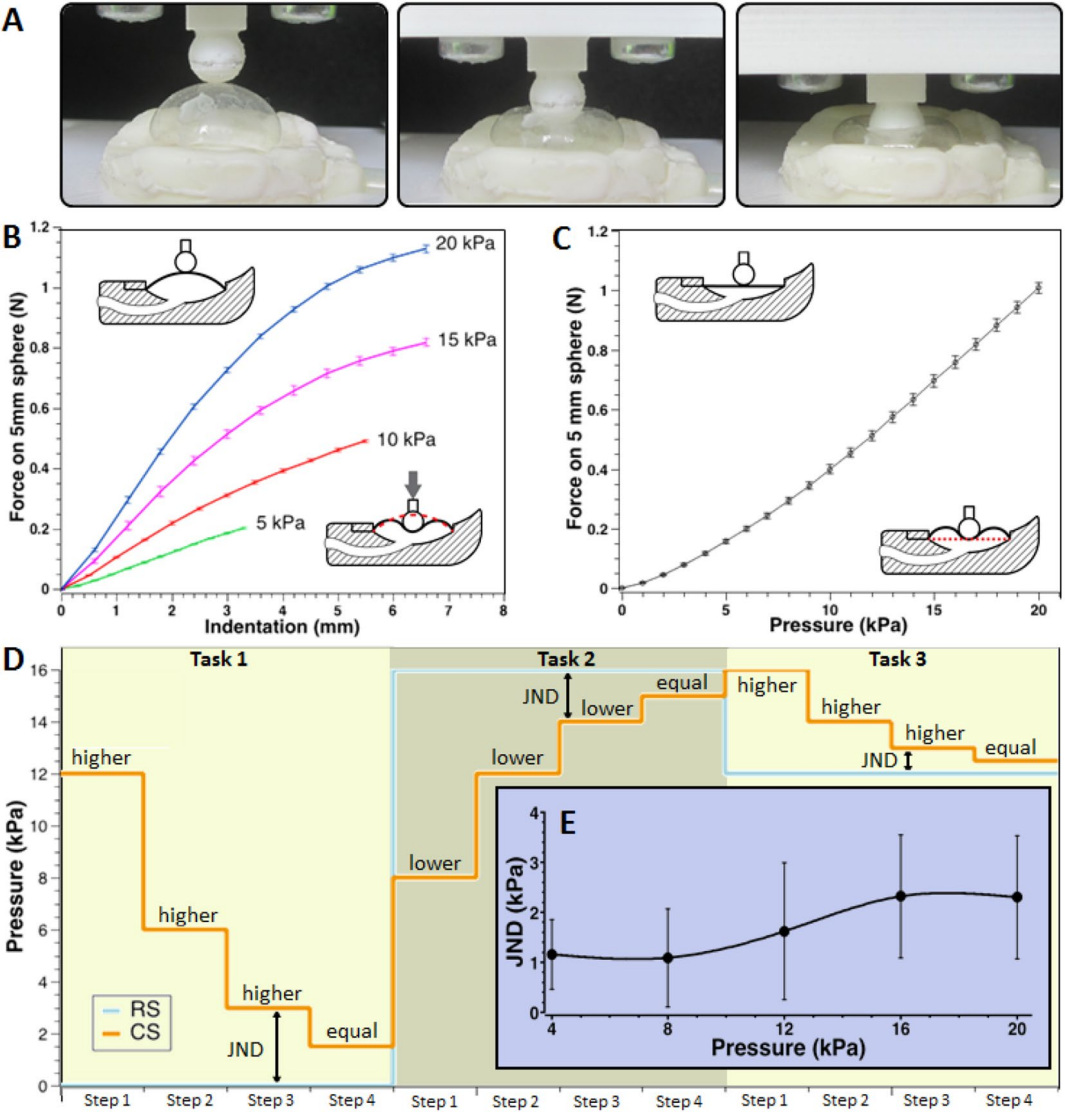
Indentation force, blocking force and psychophysical performance of the softness display. (**A**) Optical images of an indentation of the pressurised membrane with a spherical indenter having a diameter of 5 mm. (**B**) Static indentation force as a function of the indentation depth, for different driving pressures; the error bars represent the standard deviation among three samples, tested three times each. (**C**) Blocking force gathered by the same spherical indenter upon a sudden pressurisation of the membrane, as a function of the driving pressure; the error bars represent the standard deviation among three samples, tested three times each. (**D**) Example of three consecutive perceptual tasks during a psychophysical test; if a comparison stimulus (CS) is perceived as higher/lower than the reference stimulus (RS), the subsequent CS is decreased/increased until the two stimuli are perceived as equal; that final condition provides the Just Noticeable Difference (JND) related to RS. (**E**) JND as a function of the driving pressure; the error bars represent the standard deviation among ten volunteers.

In consideration of the behaviour of the membrane’s deformable surface when it is in contact with a fingertip, the objective tests were of two kinds: an indentation test, where the membrane was maintained under constant pressurisation, and a blocking force test, where the membrane was suddenly pressurised. Both these tests were performed with a spherical indenter (diameter of 5 mm) attached to a load cell, which was mounted on a micrometric three-axial translation stage. The apparatus was used as described in “Methods”. The results of the indentation and blocking force measurements at various pressures are presented in Fig. 3A–C.

The maximum force at 20 kPa was about 1 N, as measured from both kinds of tests (Fig. 3B,C). This value provides an objective quantification of the device performance, as measured in the described experimental conditions; nevertheless, it cannot be regarded as the force that would be transmitted to a fingertip in contact with the device, in response to that driving pressure. Indeed, a finger pulp is a soft body rather than a stiff sphere and its radius of curvature at the tip could be different from 2.5 mm, with variability among different individuals. This evidence leads to some considerations about modelling, which are reported later on in the text. Moreover, it highlights the importance of complementing such an objective testing with a subjective one, in order to investigate the actual perceptual response on the user and thereby evaluate the efficacy of this technology. Therefore, the next section presents subjective tests in order to evaluate the device’s psychophysical performance.

### Psychophysical performance

The display was studied with a classical psychophysical experiment, referred to as Just Noticeable Difference (JND) test. It was aimed at assessing the minimum variation of step-wise driving pressure values required for a difference in tactile perception to be noticeable. The experiment involved ten volunteers (six males and four females, aged 30 ± 5). Each participant, wearing the display on the index fingertip of their dominant hand, performed a sequence of eighteen tactile perceptual tasks. Each task was aimed at determining a JND, according to the procedure described in “Methods”. As an example, Fig. 3D presents three consecutive tasks. The outcomes of the JND tests are shown in Fig. 3E.

The JND varied in the range 0.8–2.3 kPa, for driving pressures between 4 and 20 kPa. Therefore, for the highest pressure, the JND was about one tenth of the driving stimulus. The JND monotonically grew with the stimulus, consistently with the Weber’s law^41^. The Weber constant (slope of the JND curve, as obtained from a linearization over the three central points) was *k* = 0.15.

It is worth noting that this performance was related to step-wise pressure signals (rise time lower than 0.4 s). However, if an application scenario would require pressure changes at a slower rate, it is reasonable to expect larger JND values. Indeed, due to the known frequency-dependent sensitivity of tactile receptors, it can be possible that a given force variation becomes unperceivable when presented at a very slow rate of variation, even if it is clearly detectable when delivered as a sharp change.

### Future developments: challenges towards softness control

Following this characterisation of the system, future developments should target the next important goal: the development of a strategy to accurately control the contact area and, therefore, also control the softness that can be displayed. Indeed, like any other wearable tactile display of softness described so far, this device can change the contact area only in open-loop mode. To achieve closed-loop controllability, two approaches can be envisaged: a model based control and a sensing based control, as discussed below.

The former would require an accurate physical model, able to capture the complexity of the contact mechanics at the interface between the deformable display and deformable fingertip. This is a problem dealing with finite inflations of a visco-hyper-elastic circular membrane against a soft and adhesive substrate (finger pulp), which is visco-hyper-elastic too. Such a problem can be addressed with numerical investigations, extending for instance approaches analogous to that described in^42^. Addressing this problem thoroughly in all its complexity, possibly avoiding simplistic approximations, is necessary, in order to shed light on the actual roles of the visco-hyper-elastic and geometrical parameters of both the finger pulp and the membrane. Especially, the model should take into account the variability of the mechanical parameters within the concerned deformation and frequency ranges. Let us make an example. Dhong et al. recently suggested that, according to their experiments, humans may have (perceptual) compensations for the finger deformability while judging softness, such that the finger could be considered as rigid^15^. Nevertheless, from a physical standpoint, the finger pulp has a variable stiffness, which increases for increasing compressions; so, it might be possible that an assumption of rigidity could be more or less accurate, according to the experimented indentation range.

The availability of an accurate model would then raise another challenge: how to deal with the variability of the visco-hyper-elastic and geometrical parameters of fingers across different individuals. As measuring those parameters for any new user would not be practically viable, an option could be, for instance, to take average numbers from a statistically significant population and study their accuracy for controlling the contact area. For the sake of a model validation, the area could be measured, for instance, by covering the membrane with a dye and measuring the stain on the finger, or, more accurately, using a video camera if the membrane is transparent.

In order to avoid such a complexity of a model based control, a sensing based control is preferable. However, no sensing technology appears today sufficiently mature to be integrated into an elastomeric membrane for contact area detection with high spatial resolution. Even the recent remarkable description by Sonar et al. of a fingertip-mounted pneumatic chamber with self-sensing properties unfortunately does not solve the problem, as it was based on a single sensing variable, represented by the resistance of a surface-distributed deformable conductor^38^. Indeed, by applying such a piezo-resistive sensing to the tactile display described here, it would not be possible to uniquely associate the variable resistance to the contact area, as different areas could correspond to the same resistance. This is due to the variability of both the shape and the deformability of fingertips among different individuals. Similarly, a piezo-capacitive sensing based on a single variable would not be sufficient. Actually, any strategy to detect the contact area via stretchable elastomeric resistors or capacitors would require a dense array of miniature tactile elements (“tactels”). This complicates both the manufacturing of the array and the routing of the required stretchable electrical connections to read each tactel.

In general, the problem of a sensing area reduction to allocate space for connections in an array of stretchable sensors is currently addressed with ongoing research on new reading strategies. For instance, Xu et al. have proposed that an array of elastomeric small capacitors and related connections can be replaced by a stack of two elastomeric capacitive membranes^43^; a ‘virtual’ partition of each membrane into multiple sensing elements is achieved with a multi-frequency capacitance reading^43^. This approach advantageously avoids the need for a physical addressing of each equivalent sensing element. Nevertheless, obtaining high resolutions appears challenging, due to the small differences of capacitances to be resolved between adjacent elements, as their equivalent size reduces^43^.

An alternative strategy to detect the contact area with high resolution could be offered by optical tactile sensing^44^. In general it uses one or more video cameras inside an elastomeric enclosure, to detect deformations of its surface due to contacts with an external body. Successful implementations have been demonstrated for robotic systems, especially using soft membranes internally coated with markers^44^ or a reflective layer^44,45^. Applying this sensing strategy to a wearable tactile display would require taking images from the inside of the small chamber. Therefore, it will be necessary to accommodate inside the chamber a miniature optical sensor, a wide-angle glass lens and some LEDs for illumination. Whilst the lens’ wide angle requirement would be consistent with a need for a relatively small depth of field (as the membrane in contact with a fingertip has a limited range of displacement), clearly such a video capture technology would disadvantageously increase the size of the structure, the complexity of the whole system, as well as its costs, due to the need for miniaturised components.

Therefore, according to this state of the art, future developments should necessarily address the need for a compatible sensing technology for contact area monitoring. The resolution to be achieved is currently unknown. Indeed, despite the fact that in finger pads the tactile resolution can be as low as about 0.3 mm, due to tactile hyperacuity^46,47^, it might be possible that controlling the contact area with a lower accuracy is sufficient for adequate renderings of a virtual softness. So, specific investigations are needed to find a suitable trade-off between perceptual requirements and technological limitations.

## Conclusions

We presented a fingertip-mounted tactile display to mimic non-vibratory interactions with virtual soft bodies. A custom-designed, compact and low-cost electro-pneumatic driving unit made this tactile-feedback technology advantageously portable, affordable and easily usable.

The whole system was conceived to be as simple as possible and as cheap as possible, and also free to use by anyone. This was aimed at facilitating the use of softness displays in a variety of possible applications, which at present are limited (if not prevented at all) by the practical lack of viable solutions. For this reason, the system was developed using only off-the-shelf materials and components, and without any special manufacturing process. Moreover, the architecture is here fully disclosed in all its parts, providing CAD drawings, schematics and lists of components as “Supplementary Information”. This system and its possible future developments with integrated contact-area sensing could make tactile displays of softness on fingertips a technology ‘at fingertips’.

## Methods

### Elastomeric membrane

The membrane consisted of polydimethylsiloxane (PDMS) and was purchased as a finished product (Elastosil membrane) from Wacker, Germany. It had a thickness of 70 µm.

### Manufacturing of the display

The two constitutive plastic parts of the display were 3D printed according the CAD drawings provided as .stl files in the “Supplementary Information 3”. The two parts were coupled, constraining between them the elastomeric membrane, which was arranged without any pre-stretch. A room-temperature-vulcanising silicone was used to fix two parts together (without screws, to simplify the structure) and seal the chamber.

### Static free stroke characterisation

A LASER transducer (optoNCDT ILD 1402-5, Micro-epsilon, Germany) was used to measure the unloaded membrane’s apical displacement in response to step-wise pressures at various amplitudes: 5, 10, 15 and 20 kPa. Higher values were not tested, to avoid the risk of rupture of the membrane. After the onset, the pressure was maintained constant for a few seconds, until the displacement reached a stable value, which was recorded as the free stroke. An example of static free stroke signal is shown in the Supplementary Fig. S1. After the measurement, the pressure was reverted to 0 and the test was repeated with a different pressure value.

For each considered pressure, three samples of the display were tested and, for each of them, the test was repeated three times.

### Dynamic free stroke characterisation

The LASER transducer mentioned above was also used to measure the unloaded membrane’s apical displacement in response to square pressure waves.

The motivation for using square rather than sinusoidal signals was as follows. According to the time required by the microcontroller to complete a single iteration loop, the minimum refresh time was set to 50 ms. Therefore, the refresh (sampling) frequency of the signals that could be generated was 20 Hz. This implied that sinusoidal pressures could have been generated at a maximum frequency that theoretically was limited by the Nyquist frequency of 10 Hz but practically could not exceed just a few Hz. Therefore, we opted for driving the system with square waves (on–off switching cycles), which allowed for a maximum frequency of actually 10 Hz, fully exploiting the maximum refresh rate.

The square pressure waves had an amplitude of 20 kPa and a frequency which varied in the range 0.1–10 Hz. Examples of dynamic free stroke signals are shown in Fig. 2B. For each considered frequency, three samples of the display were tested and, for each of them, the maximum and minimum values of the apical displacement signal were extracted.

### Indentation test

The test was performed as follows. The display was firstly pressurised at a given pressure and then the translation stage was moved, so as to bring the spherical indenter in gentle contact with the membrane’s apex, as detected from the load cell read-out indicating a nearly null contact force. From this set point, the translation stage was moved to indent the pressurised membrane, while keeping the pressure constant. Ten indentation steps were progressively applied, so that the membrane’s central point was taken approximately down to the position corresponding to a completely deflated device. At each step, the indentation was maintained constant for a few seconds, until the force reached a stable value, which was recorded as the force corresponding to that indentation.

This test was performed at various pressures: 5, 10, 15 and 20 kPa. For each pressure, three samples of the display were tested and, for each of them, the test was repeated three times.

### Blocking force characterisation

The apparatus used for the indentation test was also used for a blocking force test, which was performed as follows. From the rest condition of a fully deflated display (flat membrane), the spherical indenter was brought in gentle contact with the membrane’s central point, as detected from the load cell read-out (nearly null contact force). While keeping the spherical tool fixed in that position, the load cell was used to measure the membrane’s force in response to step-wise pressures at various amplitudes, ranging from 0 to 20 kPa.

At each step, the pressure was maintained constant for a few seconds, until the force reached a stable value, which was recorded as the blocking force. After the measurement, the pressure was reverted to 0 and the test was repeated with a different pressure value.

For each considered pressure, three samples of the display were tested and, for each of them, the test was repeated three times.

### JND test

The testalgorithm fixed a reference stimulus (RS), corresponding to a given driving pressure, randomly selected among a pre-defined set of values (0, 4, 8, 12, 16 and 20 kPa). The subject was stimulated with RS and, immediately after, with a comparison stimulus (CS), which was randomly selected from the remaining values of the same set. Both RS and CS consisted of step-wise pressure signals (pressure rise time lower than 0.4 s). The subject had to indicate whether CS was perceived as higher or lower than RS, or if no noticeable difference could be appreciated. Following the evaluation, the test proceeded by presenting a new couple of stimuli, where RS was unchanged and CS was modified according to the following criterion: if the previous CS had been perceived as higher/lower, the new intensity was decreased/increased by a half of the difference between CS and RS. This iterative procedure continued until the subject did not appreciate any noticeable difference; at that point, the difference between RS and CS of the previous iterative step was defined as the JND corresponding to RS. Afterwards, a new perceptual task started, with a new RS. The tests were performed in compliance with Ethical requirements for the selection of volunteers.

## Supplementary information

Supplementary Information 1.

Supplementary Information 2.

Supplementary Information 3.
