# Supplementary material for: Tactile display of softness on fingertip
Source: Sci Rep. 2020 Nov 24;10:20491. doi: 10.1038/s41598-020-77591-0 (PMC7686500; doi:10.1038/s41598-020-77591-0)
Supplement: Supplementary file 1 — Supplementary Information 1. [file 41598_2020_77591_MOESM1_ESM.pdf]

Tactile display of softness on fingertip

Gabriele Frediani, Federico Carpi

SUPPLEMENTARY INFORMATION

This PDF file includes:

Supplementary figures:
- Figure S1: Free stroke response to a pressure step of 10 kPa.
- Figure S2: Schematic diagram of the electro-pneumatic control unit.

Supplementary text.

Other Supplementary Information for this paper includes:

Files with CAD drawings of the plastic case:
- 'Display_top' (.stl format).
- 'Display_bottom' (.stl format).

File with the electronic schematic diagram:
- 'Electronic schematics' (.pdf format).

SUPPLEMENTARY FIGURES

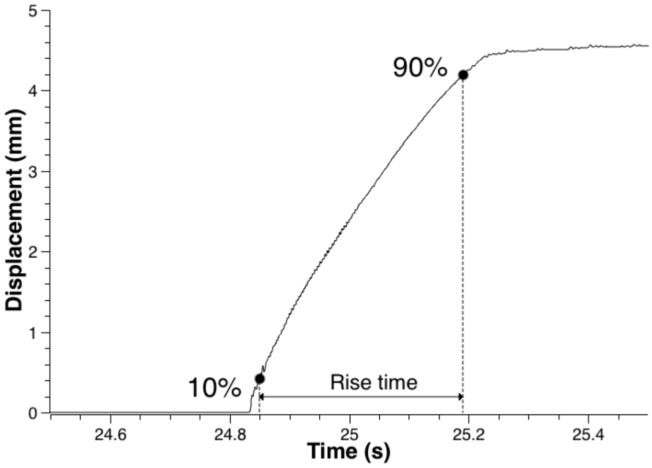

Figure S1. Free stroke response to a pressure step of 10 kPa. The rise time is calculated as the time required to displace the membrane's apex from 10% to 90% of the steady-state free stroke.

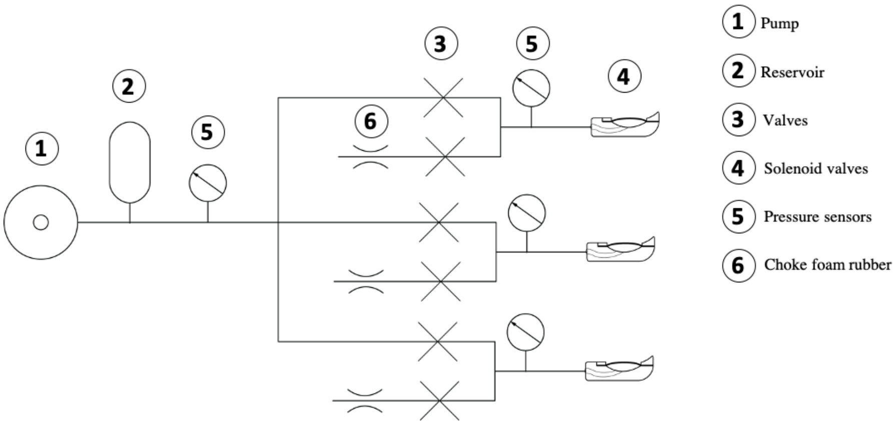

Figure S2. Schematic diagram of the electro-pneumatic control unit.

SUPPLEMENTARY TEXT

Electro-pneumatic control unit

Pneumatic connections

A schematic of the pneumatic connections is shown in Figure S2.

The pressure is provided by a rotary diaphragm pump (1) connected to a reservoir (2) used to compensate pressure fluctuations in case of sudden air consumption. Six normally closed solenoid valves (3) are used for the inflation and deflation of three displays (4). The display and reservoir pressures are measured with integrated silicon pressure sensors (5). Thin elastomeric tubing and plastic couplers are used to connect the displays to the unit.

Electronic schematics

The schematic diagram of the electronic connections is available as a separate file.
The pneumatic unit is controlled by an Arduino UNO microcontroller board.
Valves, sensors and microcontroller are connected via a custom PCB adapter.
The electronics is powered by an external 9V power supply (not shown in the schematics). Valves and pump require driving voltages of respectively 6 and 3 V, which are provided by two DC/DC converters.
Since the pump and each valve are controlled by a digital output of the microcontroller, a MOSFET is used to provide the required power.
A flyback diode is connected in parallel to the pump and each valve to eliminate flyback voltage spikes.

List of electrical and mechanical components

**Description**	**Manufacturer**	**Model**	**Q.ty**
Microcontroller	Arduino	UNO	1
Rotary diaphragm pump	Koge Micro Tec Co	KPM12C	1
Normally closed valve	Koge Micro Tec Co	KSV2WA-6A	6
Integrated silicon pressure sensors	Freescale Semiconductors	MPX5050	4
Power MOSFET	Fairchild Semiconductor	FQP30N06L	7
DC/DC Converter	Murata Power Solutions	OKI-78SR-3.3	1
DC/DC Converter	Murata Power Solutions	OKI-78SR-5	1
Diode	Vishay	SB1H100	7
Resistor	--	1 kΩ	7
Resistor	--	100 kΩ	7

Pressure control strategies

The exhaust speed of the valves was too high to allow for a fine control of the pressure provided to the displays. Therefore, it had to be reduced in order to decrease the smallest attainable change in pressure at each iteration of the control loop. To this end, two different strategies were used for inlet and outlet valves, as detailed in the following.
Inlet valves were driven by the pulse width modulated (PWM) signal provided by the digital output pins of the microcontroller. Indeed, it was found that the air flow through the valve could be modulated by changing the duty cycle of the PWM signal.
The PWM value was controlled by a linear feedback, in which the PWM duty cycle was proportional to the difference between the desired pressure and the measured pressure.
The PWM driving was not effective to drive the outlet valves. This might be due to the different pressure drop across the valves, which caused different responses to the PWM. Therefore, the outlet valves were driven in on-off mode (completely open or closed) and the reduction of the air flow was obtained by placing a foam rubber directly in the output nozzle, so as to increase frictional losses.
This simple control strategy was found to be effective. However, the open architecture of the whole system will enable, in future, the development of different control algorithms (such as PID controllers).
