# Supplementary figures and images for: Tactile display of softness on fingertip
Source: Sci Rep. 2020 Nov 24;10:20491. doi: 10.1038/s41598-020-77591-0 (PMC7686500; doi:10.1038/s41598-020-77591-0)

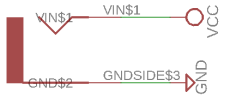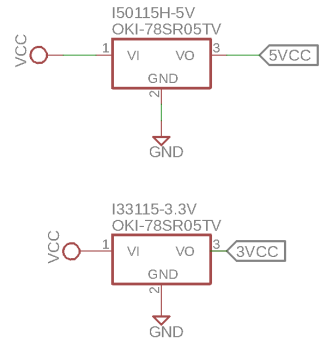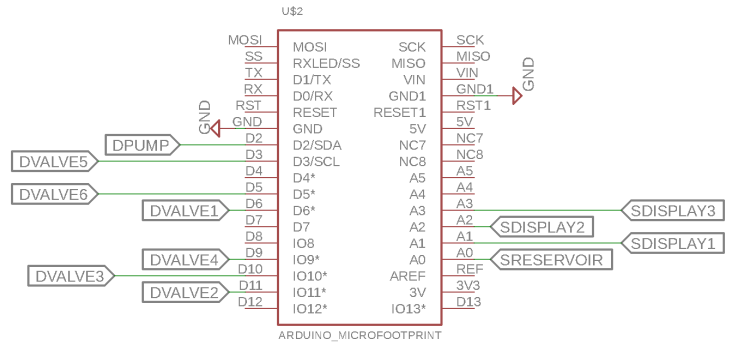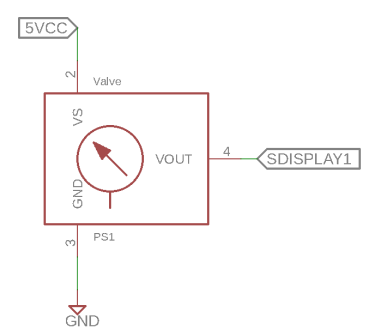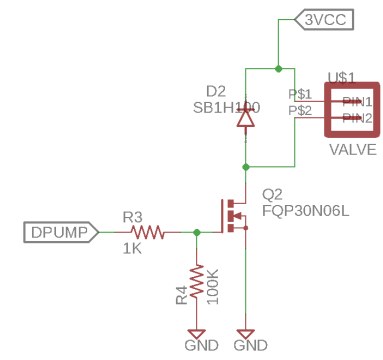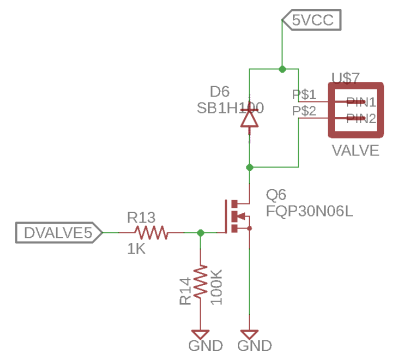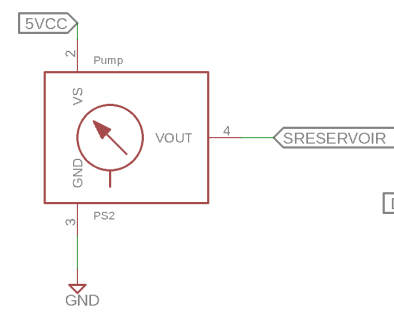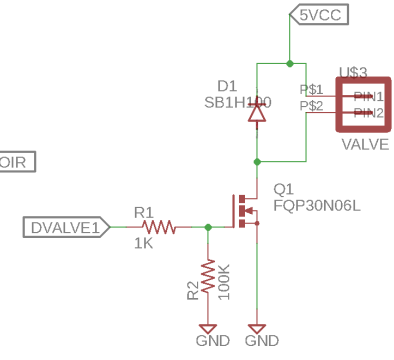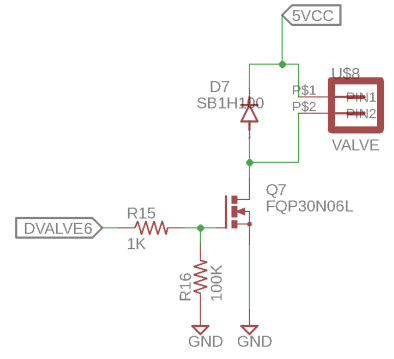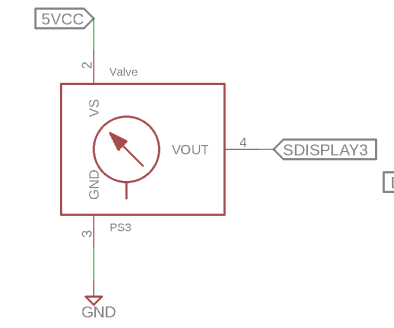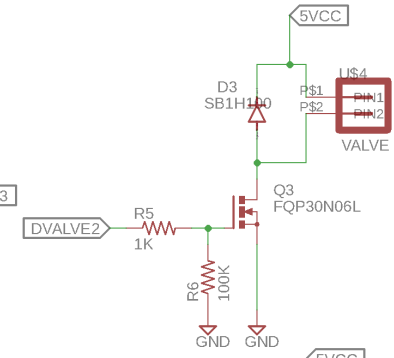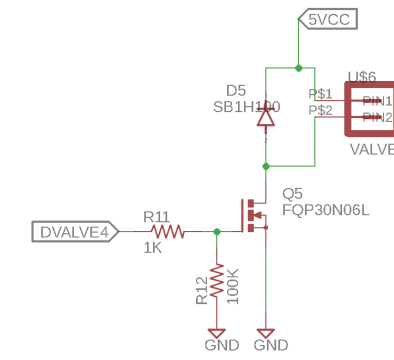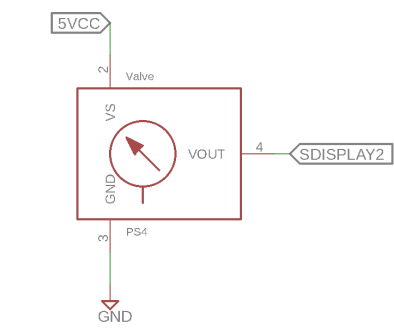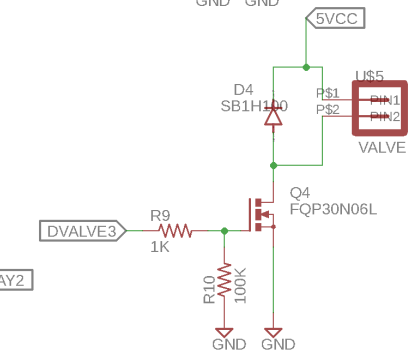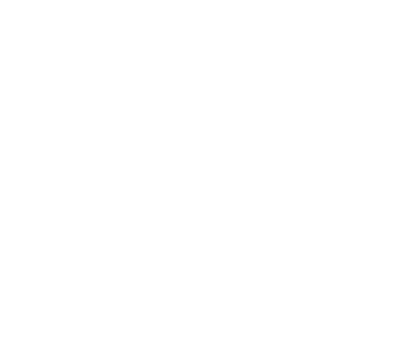

Supplement: Supplementary file 2 — Supplementary Information 2. [file 41598_2020_77591_MOESM2_ESM.pdf]
